# When Patent Foramen Ovale (PFO) Can Cause Trouble—A Misplacement of Pacemaker Lead Into the Left Ventricle

**DOI:** 10.1155/cric/6816373

**Published:** 2026-01-15

**Authors:** Ayman Helal, Ibrahim Antoun, Mohammed El-Din, Mohsin Farooq

**Affiliations:** ^1^ Department of Cardiology, Kettering General Hospital, University Hospitals of Northamptonshire, Kettering, Northamptonshire, UK, nhs.uk; ^2^ Department of Cardiovascular Science, University of Leicester, Leicester, Leicestershire, UK, le.ac.uk

**Keywords:** case report, lead malposition, left ventricular pacing, pacemaker complication, patent foramen ovale (PFO)

## Abstract

Misplacement of pacemakers lead into the left ventricle (LV) is a rare but clinically important complication, often facilitated by unrecognized intracardiac shunts such as a patent foramen ovale (PFO). Early recognition is essential to avoid systemic embolization and ensure safe device function. We report a man in his 70s with a background of bioprosthetic aortic valve replacement, coronary bypass grafting, hypertension, chronic kidney disease, Parkinson′s disease, and prostate cancer, who underwent permanent pacemaker implantation for symptomatic sinus pauses. Follow‐up echocardiography 1 year later, performed as part of surveillance of his aortic valve prosthesis, unexpectedly revealed that the ventricular lead had crossed a PFO and was positioned in the LV via the mitral valve. His 12‐lead ECG demonstrated a right bundle branch block‐like paced morphology, raising suspicion of LV pacing. The patient remained asymptomatic with no evidence of systemic embolization. He was anticoagulated with apixaban and subsequently underwent successful lead extraction and repositioning into the right ventricle (RV). Correct RV placement was confirmed using multiple fluoroscopic views, particularly the left anterior oblique (LAO) projection and by postprocedure ECG, chest x‐ray, and echocardiogram. This case underlines the importance of careful assessment of paced ECG morphology, fluoroscopic views during implantation (especially LAO), and postimplant imaging to confirm lead location. Suspicion should be raised when an RBBB‐like QRS morphology is observed during RV pacing. Timely recognition and management with anticoagulation, followed by extraction and repositioning, can prevent potentially devastating complications. Operators should remain vigilant for inadvertent LV lead placement, particularly in patients with unrecognized PFO. Routine use of multiple fluoroscopic projections and correlation with ECG and echocardiography can aid early diagnosis and improve procedural safety.

## 1. Introduction

Patent foramen ovale (PFO), a remnant of fetal circulation, can occasionally result in significant clinical complications when undiagnosed. In this case, we report a patient in his 70s who had a pacemaker lead unintentionally placed in the left ventricle (LV) through a PFO instead of the right ventricle (RV). This rare occurrence underscores the importance of recognizing specific clinical and diagnostic signs during and after pacemaker implantation. We also discuss how to detect such misplacements early and present the patient′s management course, including lead extraction and repositioning.

### 1.1. Case Presentation

**Figure 1:**
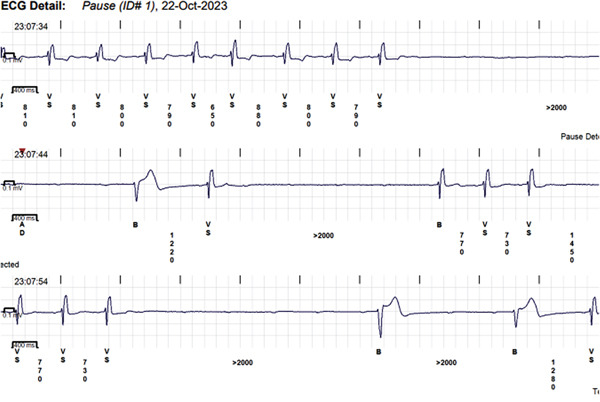
Sinus pauses detected by an implantable loop recorder.

A patient in his 70s with a significant past medical history, including bioprosthetic aortic valve replacement (25 mm Hancock valve) and bypass graft (saphenous venous graft [SVG] to right coronary artery [RCA]) 7 years prior to presentation; additional comorbidities include hypertension, dyslipidemia, chronic kidney disease (CKD) Stage 3, idiopathic Parkinson′s disease, and prostate cancer treated with radiotherapy. He had pacemaker implantation 1 year prior to presentation. The pacemaker was indicated for syncope, which was correlated to sinus pauses by an implantable loop recorder Reveal LINQ device, Medtronic (Figure 1), which demonstrated ventricular standstill of 5–6 s.

**Figure 2:**
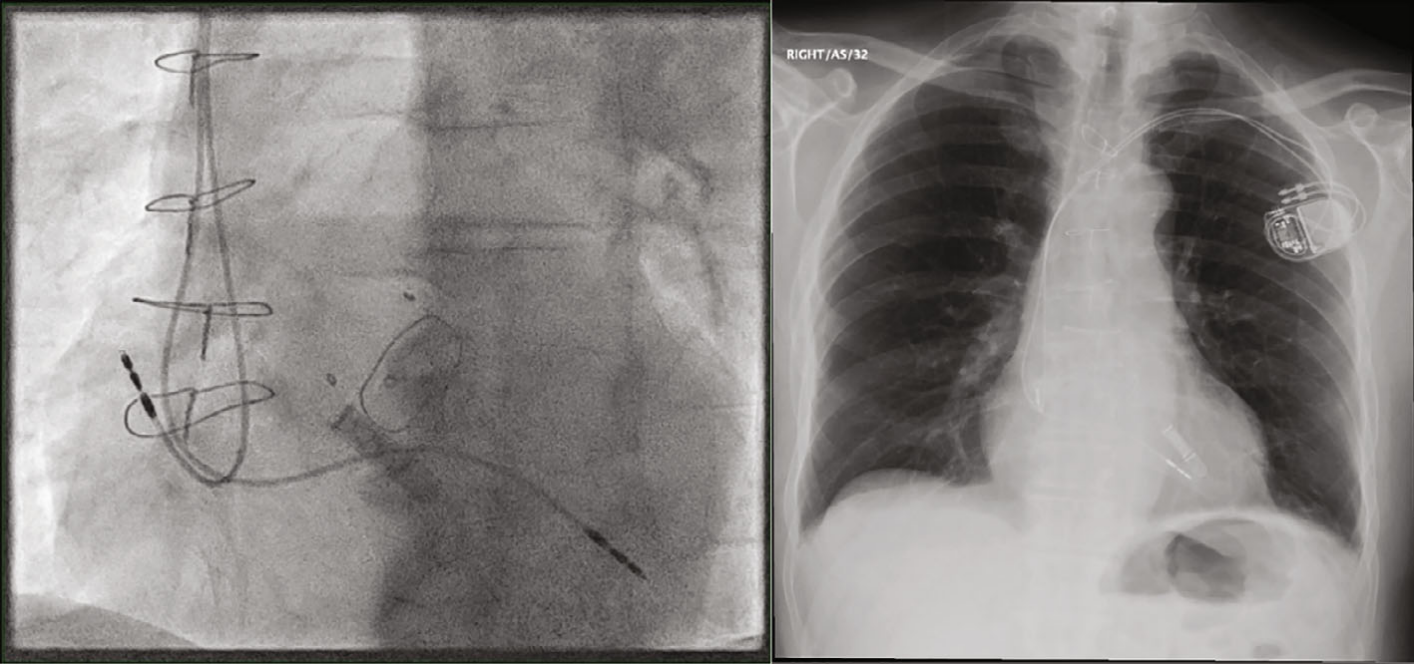
Confirmation of the pacemaker leads placement by fluoroscopy and chest x‐ray.

He underwent successful pacemaker implantation (Biotronik, Enitra 6 DR‐T), with a right atrial (RA) lead placed in the right atrium (Biotronik, Solia Pro MRI S 53, Bipolar) and the right ventricular (RV) lead intended for the RV apex (Biotronik, Solia Pro MRI S 60, Bipolar) (Figure 2).

However, the follow‐up echocardiography (indicated for the bioprosthetic valve annual follow up) 1 year postdevice implantation revealed that the RV lead had passed through the PFO and was positioned in the LV, crossing from the right atrium (RA) to the left atrium (LA) and then through the mitral valve into the LV (Figure 3).

Figure 3Echocardiogram showing the ventricular lead crossing (yellow arrow) from the right atrium to left atrium through PFO then to left ventricle in different views: parasternal long axis (a), apical four‐chamber view (b), subcostal (c) views with colored Doppler imaging showing mild mitral regurgitation (d).

**Figure:**
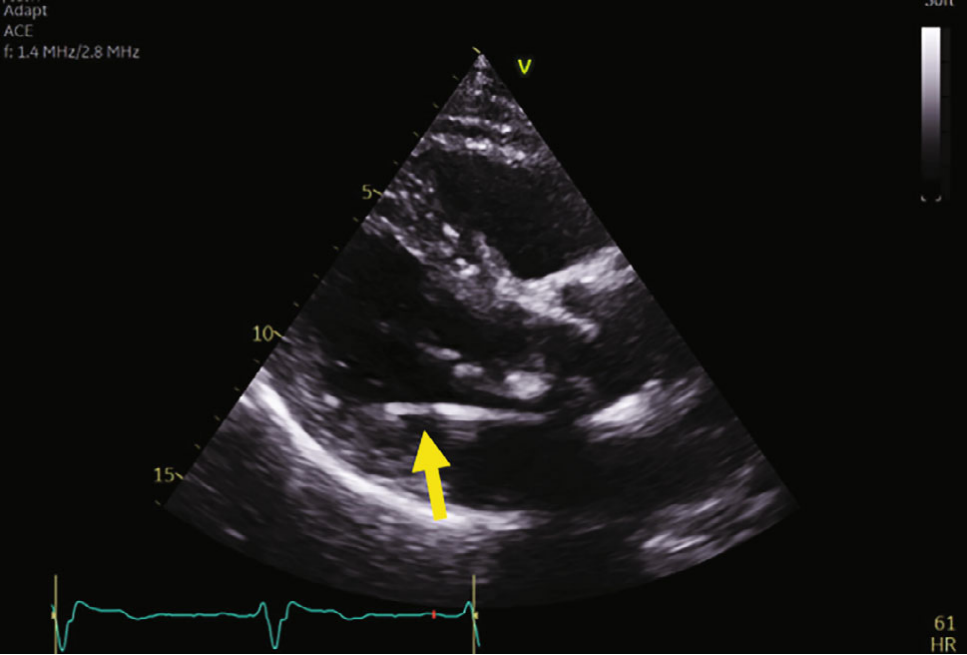
(a)

**Figure:**
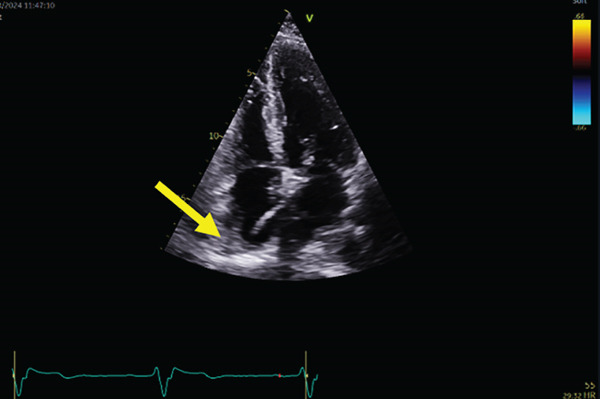
(b)

**Figure:**
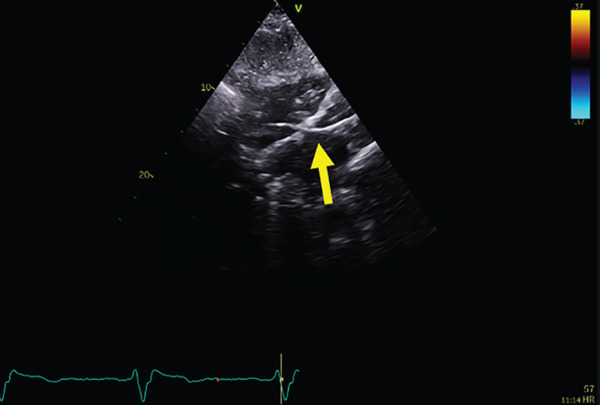
(c)

**Figure:**
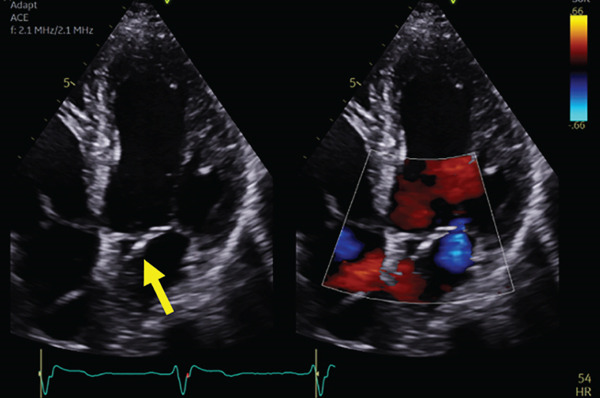
(d)

**Figure 4:**
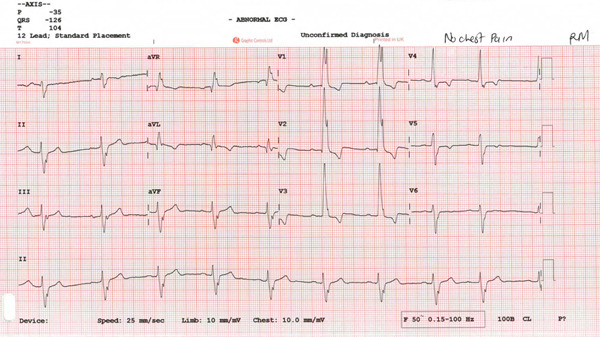
Patient′s 12 leads ECG demonstrated right bundle branch block (RBBB) morphology of the paced QRS complex with an axis of −120° denoting pacing from the left ventricle.

**Figure 5:**
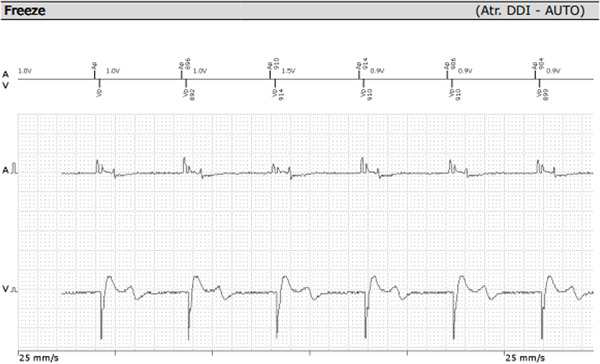
Satisfactory pacemaker checks.

The patient remained asymptomatic since implantation, with normal biventricular function. His 12 leads ECG (which was performed following the echocardiogram to assess the device) clearly demonstrated right bundle branch block (RBBB) morphology of the paced QRS complex with an axis of −120° (Figure 4) denoting pacing from the left ventricle. The pacemaker device download did not demonstrate any arrhythmia; his pacemaker checks were all satisfactory (Figure 5).

There was no clinical evidence of systemic embolization, but we started anticoagulation with apixaban, and we decided to proceed with lead extraction and replacement to correct this complication.

### 1.2. Management and Follow‐Up

**Figure 6:**
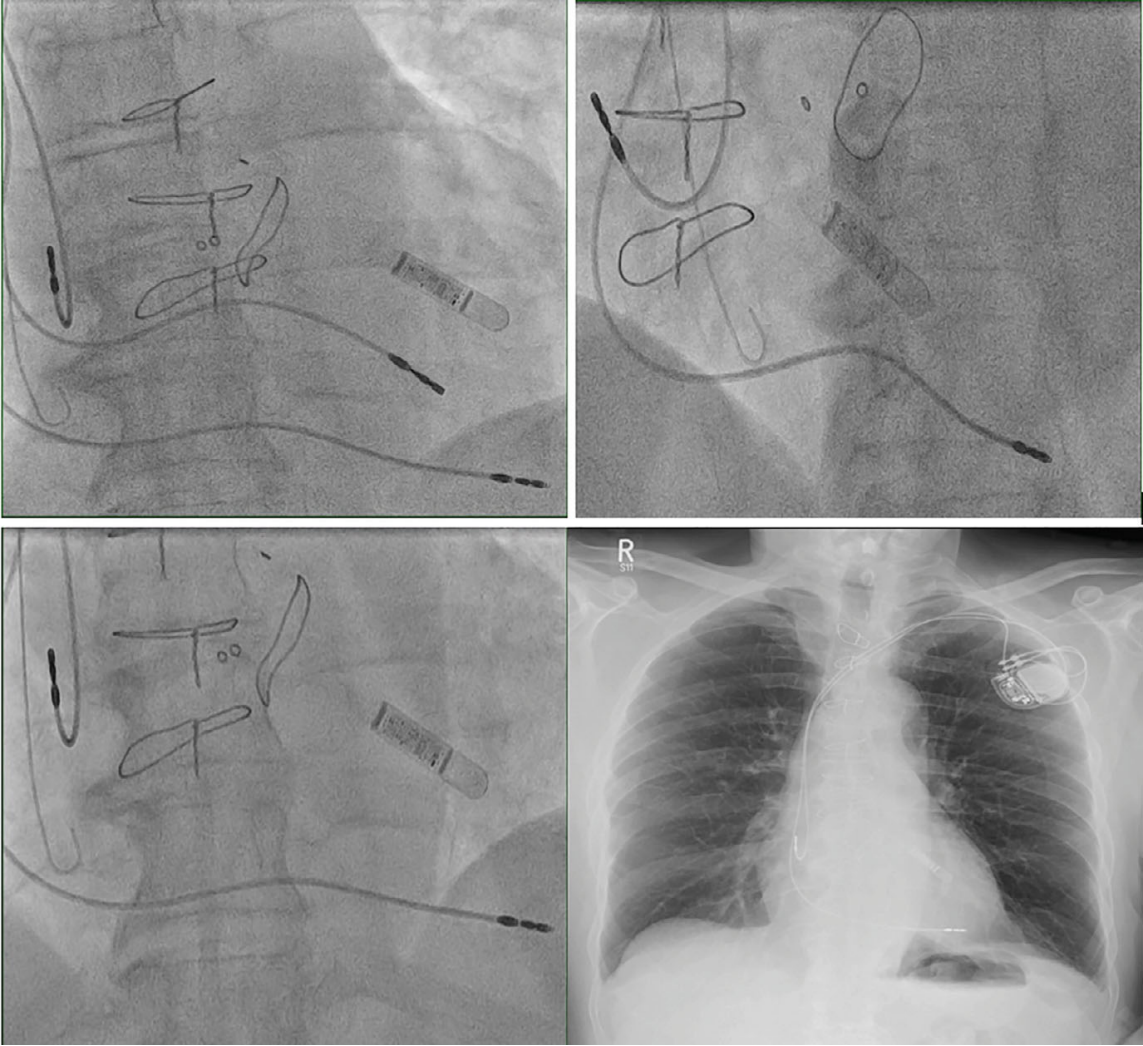
Confirmation of leads position (top left: both the old LV lead and the new RV lead before LV lead extraction, top right: RV lead in LAO view, bottom left: RV lead in RAO view, and bottom right: post‐procedure chest x‐ray).

The patient underwent lead extraction and repositioning of the pacemaker lead into the RV after 1 year of the original implantation. Anticoagulation therapy with apixaban was initiated to mitigate the risk of thromboembolic complications while awaiting lead revision [1]. Aspirin was suspended during this period to reduce bleeding risk. We implanted a new RV lead; we initially crossed the lead with a large knuckle through the tricuspid valve and advanced it toward the right ventricular outflow tract (RVOT) to ensure lead placement into the RV apex. On fluoroscopy, the LV and RV leads follow different orientations (Figure 6). We confirmed position via obtaining different fluoroscopic views including RAO (right anterior oblique) and LAO (left anterior oblique), then we explanted the LV lead. An Electrocardiogram (ECG) comparison between pacing form the LV and RV leads in the presenting case are demonstrated in Figure 7. It was exceedingly difficult to differentiate in this case; however, the biphasic complexes which is usually seen in RV septal rather than apical pacing should raise the suspicion. The patient tolerated the procedure well and remained stable postoperatively. The apixaban was stopped after the procedure and aspirin was started again. Patient was sent home on the same day.

Figure 7Pacing from the LV lead (a) compared with pacing from the RV lead (b) during the new lead placement. The biphasic complexes which are usually seen in RV septal rather than apical pacing should raise the suspicion of abnormal lead position.

**Figure:**
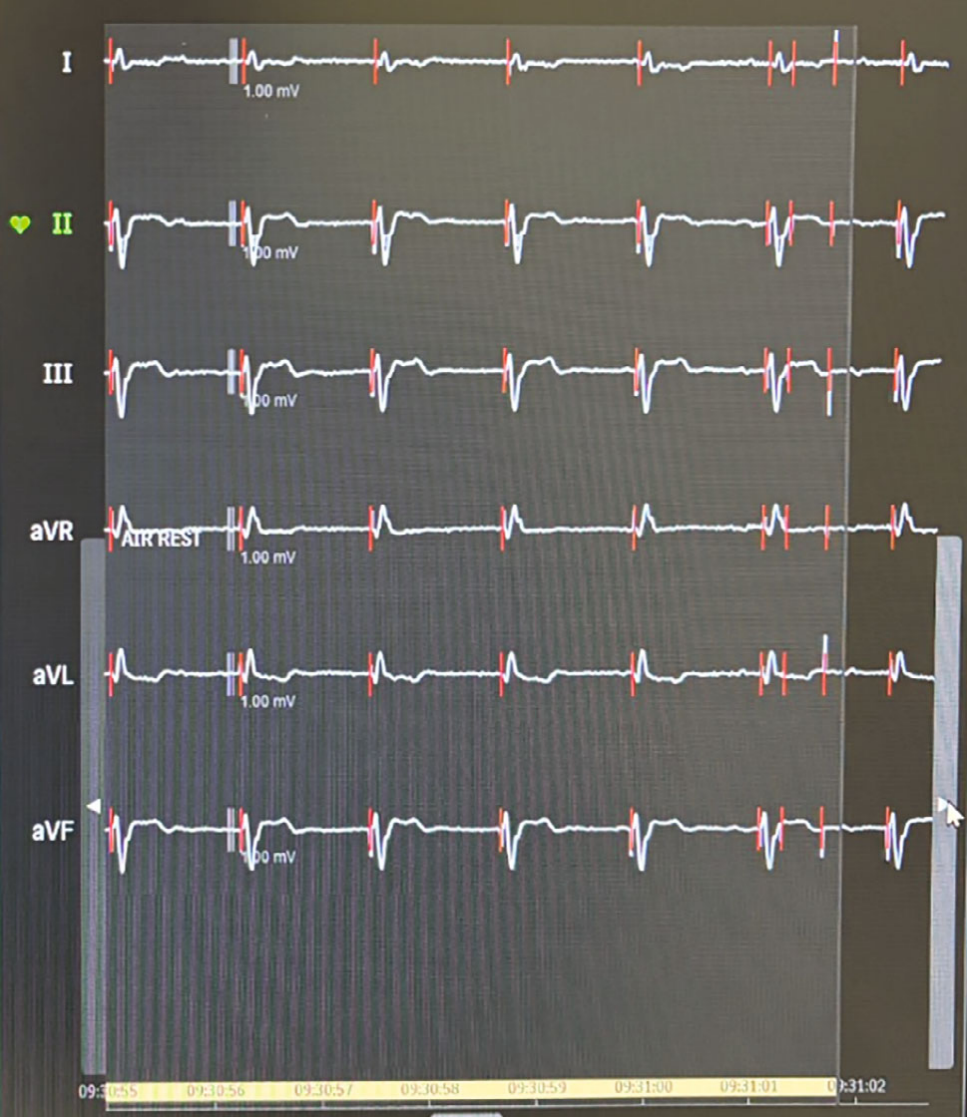
(a)

**Figure:**
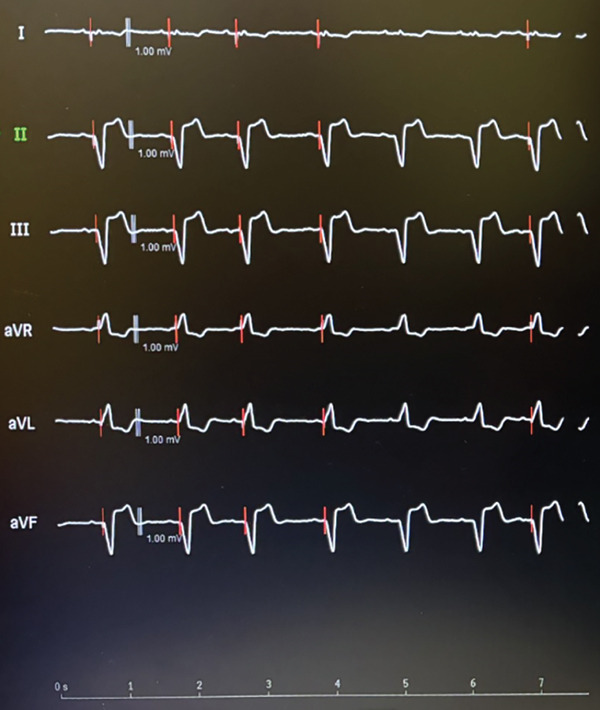
(b)

## 2. Discussion

### 2.1. How to Suspect LV Lead Misplacement

Although pacemakers′ leads misplacement into the LV is rare, its consequences can be significant, including systemic thromboembolism and impaired LV function. Early detection and intervention are critical to preventing complications [1].

Key factors that can raise suspicion of lead misplacement include abnormal pacing patterns, unexpected ECG findings, and imaging discrepancies.

1. *ECG clues*: Inappropriate lead placement can often be suspected from the ECG. When pacing from the RV, the QRS complexes are typically left bundle branch block (LBBB)‐like, with a rightward axis. However, in LV pacing, the QRS complexes may mimic RBBB with a leftward axis (Figure 8) [2]. Electrocardiographic localization of lead position that exhibits RBBB morphologies during pacing is summarized in Table 1 [3]. In the presenting case, the patient′s 12‐lead ECG showed an RBBB‐like pattern (Figure 4), indicating the possibility of LV pacing.

Figure 8ECG comparison between LV pacing in Panel a showing right bundle branch block morphology versus RV pacing in panel b which shows LBBB morphology.

**Figure:**
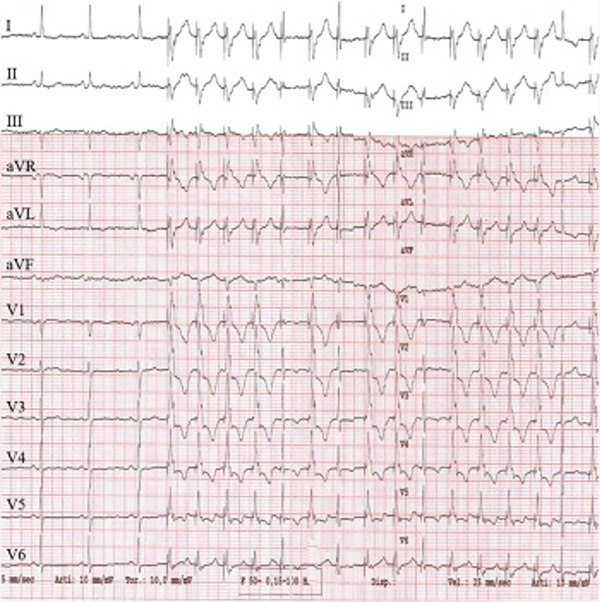
(a)

**Figure:**
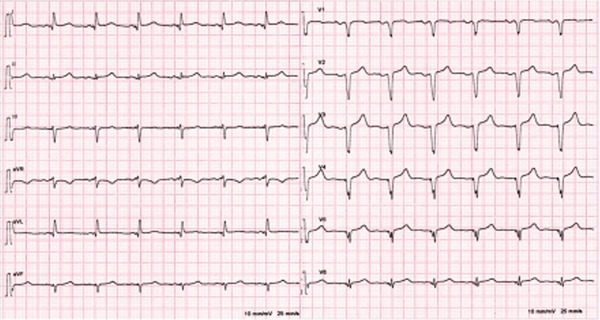
(b)

**Table 1:** Electrocardiographic localization of leads that exhibit right bundle branch block morphologies during pacing [3].

**Frontal axis**	**Precordial transition**	**Location**	**Sensitivity %**	**Specificity %**	**Positive predictive value %**
0 to −90	By V3, by V4, by V4, after V4	RV septum or apex, RV septum or apex, posterior LV or coronary vein posterior LV or coronary vein	86, 100, 26, 72	99, 92, 83, 100	95, 64, 36, 100
−90 to −180	By V3	LV apex and distal anterior LV	85	100	100
90–180		Proximal anterior and anterolateral LV	100	97	90

**Figure 9:**
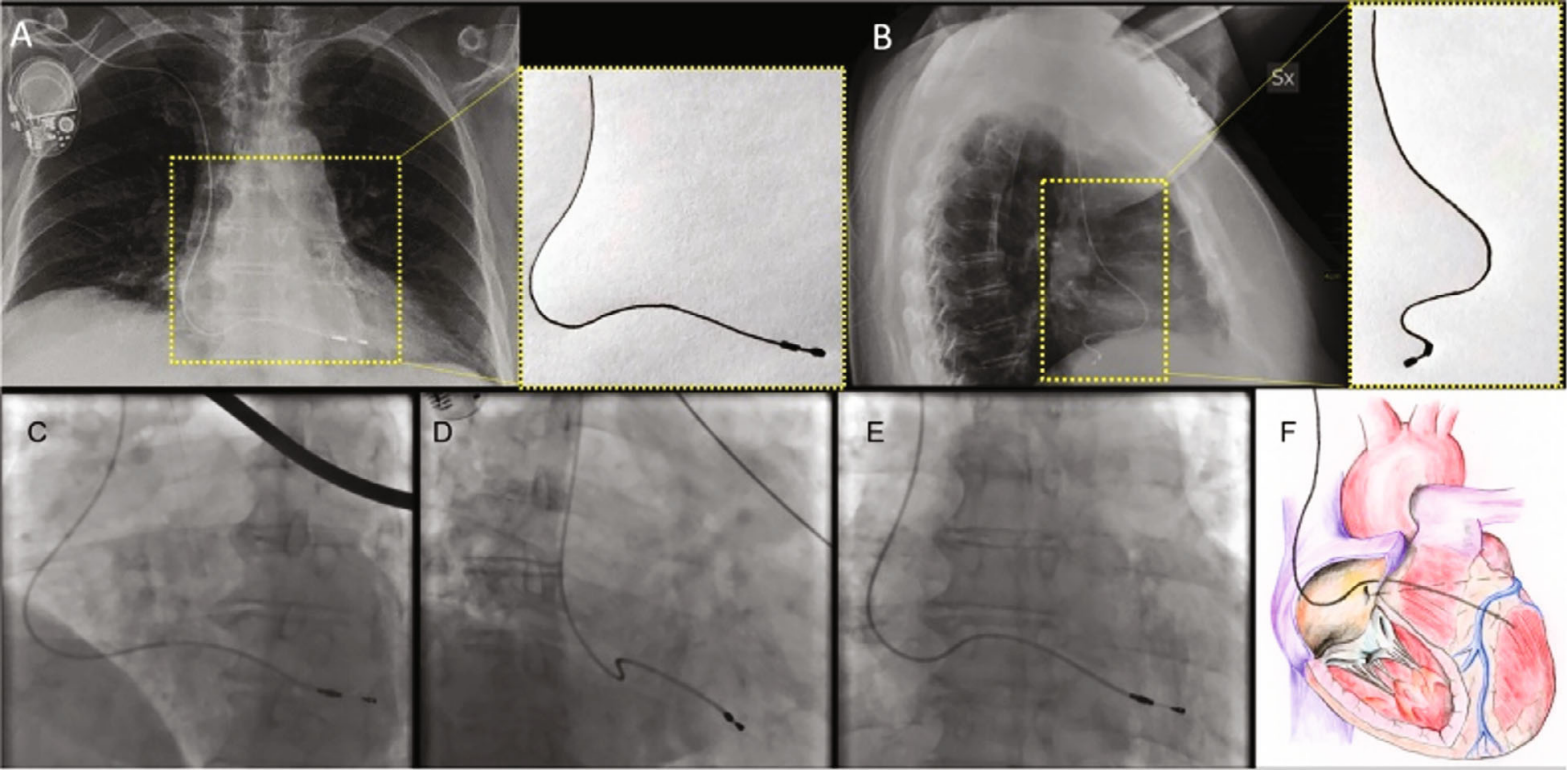
In the upper box, patient′s chest x‐ray: posteroanterior (PA) view (A); latero‐lateral view (B) with blowup indicating the pathway of the lead in the two projections; in the lower box: fluoroscopic images, 40° left anterior oblique view (C); 30° right anterior oblique view (D); PA view (E); a schematic representation of the lead malpositioned in the left ventricle, based on the PA projection; the location of the coronary sinus is depicted by the dotted line (F) [6].

2. *Fluoroscopy*: Fluoroscopic guidance during pacemaker implantation is the gold standard for lead placement. However, subtle misplacements like PFO‐related LV positioning may not be immediately apparent, especially when the lead follows an unexpected path. Careful inspection of the lead trajectory is critical [4]. The LAO view is especially useful as it provides a clear perspective on the lead′s orientation and helps differentiate between a correct RV placement and an unintended LV position through a PFO. In the LAO view, RV leads should be directed toward the RV apex, whereas leads that deviate toward the left side of the heart or cross the septum may indicate incorrect positioning in the LV. By observing the trajectory of the lead during implantation, the operator can immediately suspect mispositioning and take corrective action [5]. The importance of different fluoroscopic views is demonstrated in Figure 9 [6].

3. *Imaging*: Postimplantation echocardiography is important in confirming lead position, especially in patients with abnormal ECGs or unclear symptoms. The echocardiogram in this case clearly showed the lead traversing the PFO and entering the LV through the mitral valve. This imaging modality remains essential in identifying anatomical abnormalities contributing to lead misplacement, such as a PFO. [7] More dedicated imaging techniques can also be considered including CT and MRI to confirm the leads position [8, 9].

### 2.2. Extracting the Misplaced Lead

Lead extraction in patients with LV lead placement requires careful planning, particularly due to the risk of thromboembolism. Anticoagulation therapy before extraction is essential [9]. In this case, the patient was commenced on apixaban prior to the procedure to reduce thromboembolic risk. The extraction was performed uneventfully, and the new lead was correctly positioned in the RV. This was confirmed by fluoroscopy and postoperative ECG, chest X‐ray, and echocardiogram.

## 3. Conclusion

This case highlights the importance of thorough evaluation during pacemaker implantation, particularly in patients with anatomical variations such as a PFO. Early detection of LV lead misplacement is crucial to preventing complications, and clinicians should be vigilant for abnormal pacing patterns and imaging findings postimplantation. Anticoagulation and careful procedural planning are key to successful lead extraction and repositioning in these patients.

## Consent

Patient consent has been obtained.

## Disclosure

This work contained no materials from other sources.

## Conflicts of Interest

The authors declare no conflicts of interest.

## Funding

No funding was received for this manuscript.

## Data Availability

The data that support the findings of this study are available from the corresponding author upon reasonable request.
